# A Deliberate Choice? Exploring the Decision to Switch from Cigarettes to E-Cigarettes

**DOI:** 10.3390/ijerph16040624

**Published:** 2019-02-20

**Authors:** Kim A.G.J. Romijnders, Liesbeth van Osch, Hein de Vries, Reinskje Talhout

**Affiliations:** 1 Centre for Health Protection (GZB), National Institute for Public Health and the Environment (RIVM), Bilthoven, 3721 MA, the Netherlands; reinskje.talhout@rivm.nl; 2 Department of Health Promotion, School for Public Health and Primary Care (CAPHRI), Maastricht University, Maastricht, POB 616 6200 MD, the Netherlands; liesbeth.vanosch@maastrichtuniversity.nl (L.v.-O.); hein.devries@maastrichtuniversity.nl (H.-d.-V.)

**Keywords:** informed decision making, focus groups, qualitative research, dual use, harm reduction, smoking prevention, targeted communication

## Abstract

E-cigarettes are increasingly popular among both cigarette smokers and non-users. Although smoking cessation yields the most individual and population health benefits, switching to exclusive e-cigarette use offers some individual health benefits for cigarette smokers. However, e-cigarette use is not harmless, and its use among non-cigarette smokers should be prevented. Our study aims to explore the decision-making process about e-cigarettes among an e-cigarette users, cigarette smokers, and non-users. We conducted 12 semi-structured focus group interviews with e-cigarette users, cigarette smokers, and non-users. We performed a thematic analysis of the interview transcripts. First, knowledge reported by e-cigarette users was mainly based on other users’ experiences. Second, cigarette smokers and non-users were more negative towards e-cigarettes than e-cigarette users. Third, e-cigarette users considered switching from cigarette smoking to e-cigarette use by deliberating relevant information, and weighing up the benefits and disadvantages of e-cigarette use versus smoking. Additionally, important factors in the decision-making process were a perception of risks and benefits of e-cigarettes compared to cigarettes, a supportive social environment about e-cigarette use, and trust in information offered about the risks and benefits of e-cigarettes. Our findings provide insight into what we can learn from the conscious decision-making process of e-cigarette users who switched from cigarettes to e-cigarettes. This information can be considered to develop targeted communications strategies to stimulate a conscious decision-making process, these may highlight benefits of switching to e-cigarettes for cigarette smokers, discussing the risks of smoking, and correcting misperceptions about the perceived risks and benefits of e-cigarette use.

## 1. Introduction

Electronic cigarettes (e-cigarettes) vaporize a solution of glycerin, propylene glycol, additives, and sometimes nicotine [1,2,3,4,5]. While early models mimic conventional cigarettes (in shape, flavor, and size), the design characteristics of newer models and e-liquid flavors available are elaborate and attractive to both cigarette smokers and non-users [3,6,7,8,9,10,11,12]. In 2018, 3.1% of the Dutch population ever used an e-cigarette. In addition, 23.1% of the Dutch population were smokers, of which one-third were between twenty and twenty-four years old [13].

E-cigarettes have the potential to reduce the harm of cigarette smoking on smokers’ health [10]. Recent studies have argued that—while quitting the use of tobacco and related products yields the most health benefits—the health effects associated with exclusive e-cigarette use (and not dual use of cigarettes and e-cigarettes) are less harmful than those related to cigarette smoking [11,14,15,16,17,18,19]. Still, e-cigarette use is not harmless, and consequently, the initiation of e-cigarette use among non-users needs to be prevented [10,11,20,21,22]. To aid tobacco control that yields the most public health benefits for cigarette smokers and non-users, insight into differences in the decision-making process of e-cigarette users, smokers, and non-users is needed about e-cigarette use initiation. In particular, it is important to know whether the decision made by former cigarette smokers to switch to e-cigarettes, or by non-users to refrain from e-cigarette use, is consciously deliberated. 

Previous research among different types of users and different age groups has described differences in reasons why initiating e-cigarette use is attractive [23,24,25]. For example, cigarette smokers have argued that the expected health benefits of e-cigarette use compared to cigarette smoking and design similarities with cigarettes are reasons to initiate e-cigarette use [25,26,27]. Non-using youth (<18 years) have stated curiosity as a reason to initiate e-cigarette use [25]. Despite previous research into reasons for initiating e-cigarette use, [25,27] insight into the process of making a choice (decision-making) about e-cigarette use initiation lacks. This information is crucial to facilitate a conscious decision among cigarette smokers about switching to e-cigarettes and among non-users about refraining from experimenting with e-cigarettes.

A conscious decision-making process is defined by deliberating available and relevant information about the options, and by weighing advantages and disadvantages of the choice in the context of the decision-maker’s attitudes [28,29,30]. Previous research found that smokers have not made a conscious and informed decision to initiate cigarette smoking, but that this decision is rather passive [28,29,31,32,33,34]. Our study explores the deliberation process of successful switching among current e-cigarette users (former smokers), and the deliberation process of refraining from e-cigarette use among cigarette smokers and non-users. To the best of our knowledge, this is the first study to examine the decision-making process of e-cigarette use among cigarette smokers and non-users. We explored whether e-cigarette users, cigarette smokers, and non-users have similar knowledge and attitudes regarding e-cigarettes, and if they used this knowledge to consider pros and cons of switching to, or refraining from e-cigarette use. By comparing differences in this process of e-cigarette use initiation between e-cigarette users, cigarette smokers, and non-users, we offer new information on the views and experiences of e-cigarette users who switched from cigarettes to e-cigarettes.

## 2. Materials and Methods 

To explore the decision-making process about e-cigarette use, we conducted focus groups with three homogeneous groups consisting of adult e-cigarette users, adult cigarette smokers, and adolescent non-users who used neither cigarettes nor e-cigarettes. For each of these types of users, four focus groups were conducted. The study was approved by the Medical Ethics Committee of Zuyderland—Zuyd (16-N-84).

### 2.1. Recruitment

In June 2016, participants were recruited in the Netherlands, through an advertisement on Facebook©, Instagram©, Twitter©, and scholieren.com (a website targeted at adolescents in the Netherlands). Inclusion criteria were: awareness of e-cigarettes, being a current smoker (18+), e-cigarette user (18+), or non-user (13 to 17 years old), and fluency in Dutch. Adolescent non-users were selected because previous research showed differences in reasons for e-cigarette initiation among adult e-cigarette users, adult cigarette smokers, and adolescent non-users [25]. Supplementary file Table S1 shows the characteristics of included participants. 

### 2.2. Procedure

The group discussions were based on a semi-structured protocol with open-ended questions and minimal steering to allow participants to freely discuss e-cigarettes. [35] The topic list of this protocol was developed to investigate decision-making according to the definition of van den Berg, Timmermans, ten Kate, van Vugt, and van der Wal [29], and previous qualitative research conducted by Gray, Hoek, and Edwards [31]. This topic list was tested in a pilot focus group and subsequently revised. While the same moderator (K.A.G.J.R.) led all group discussions, the observers varied. All focus groups were audio recorded, and flipcharts were used for participants to visualize the topics that had been discussed (see Supplementary file Table S2 for a detailed summary of the semi-structured protocol).

The focus groups were conducted between June and July 2016, and lasted approximately 2 hours. Informed consent was obtained before the start of the focus group and after participation, the participants received an incentive of 30 euros. The focus group started with a general introduction. The discussion proceeded in four steps exploring participants’ knowledge regarding e-cigarettes, their attitude towards e-cigarettes, their deliberation regarding e-cigarette use initiation, and their information needs (see Supplementary file Table S2 for a detailed summary).

### 2.3. Qualitative Coding and Analysis 

All group discussions were transcribed verbatim. Qualitative data analysis comprised of three phases [36]. In the first phase, the first author applied descriptive level coding to a randomly selected focus group transcript, to deduce relevant themes and subthemes. The first author and the second coder (T.J.) then developed a descriptive thematic analysis. The second coder identified little additional text fragments to be relevant. In the second phase, the first and the second coder resolved minor differences in coding themes and subthemes to create a final coding taxonomy. A third coder (M.B.), who did not participate in the first phase of the coding process, reviewed the final coding taxonomy with the first author and minor adjustments were made. In the final and third phase, the first author and third coder coded all the transcribed focus groups.

## 3. Results

We conducted four focus groups with adult e-cigarette users (*n* = 26), four focus groups with adult cigarette smokers (*n* = 17), and four focus groups adolescent non-users (*n* = 23). All e-cigarette users reported to be former smokers. Overall, the sample was 47% female and 53% male, the average age of adult participants was 29.3 years (±13.0, min = 18, max = 58), and adolescents 15.3 (±1.1, min = 13, max = 17), and the sample was highly educated (applied sciences or university degree; 65%). Table 1 summarizes the demographic variables for e-cigarette users, cigarette smokers, and non-users. The following section describes themes, which were considered important in the overall decision-making process of e-cigarette users, smokers and non-users with illustrative quotes. Because of the explorative and qualitative nature of this study, the results are not meant to convey generalizability beyond the studied population. To describe themes and patterns in the data, we distinguish between rarely and commonly mentioned aspects [37].

### 3.1. Knowledge

When participants were asked to talk about what they knew about e-cigarettes, many participants were able to mention some facts about e-cigarettes. For example, many participants stated the variety of e-liquid flavors available, the rechargeable batteries of e-cigarettes, the lack of a burning process and tar, the variety of devices, and the ability to avoid smoking bans (quote 1).

*‘You use an e-liquid, there are a lot of flavors, you can choose whether you want with or without nicotine, and there are many different types…’*

*(#3_smoker about e-cigarettes)*

Compared to cigarette smokers and non-users, e-cigarette users described more detailed information about e-cigarettes. E-cigarette users reported to have acquired much information in order to make a decision about e-cigarette use initiation, for example, about product characteristics, ingredients of e-liquids, and legislation regarding e-cigarettes. Knowledge reported by e-cigarette users was mainly based on user experiences. When asked how informed they felt, cigarette smokers and non-users stated that they did not search for information about e-cigarettes and expressed they did not know much about e-cigarettes. E-cigarette users felt very informed.

### 3.2. Attitude 

Participants shared experiences, both positive and negative, with e-cigarettes and cigarettes. Cigarette smokers and non-users, were negative towards e-cigarette use, in general mentioning it was ‘weird’ (quote 2):

*‘I think it’s [e-cigarettes] really weird and pathetic. Everybody will be laughing at you.’*

* (#23_non-user)*

All groups mentioned that using e-cigarettes has the advantage of avoiding smoking restrictions (quote 3).

*‘It [e-cigarettes] is really easy to use […] and you can even use it inside, great if you’re in the bus […] Or you can use it for a just bit, instead of smoking an entire cigarette. I think I’d enjoy that…’*

* (#17_smoker)*

Compared to e-cigarette users and non-users, many cigarette smokers were positive towards cigarette smoking. Regardless of the negative health effects associated with cigarette smoking, smokers expressed they really enjoy smoking. Several of the e-cigarette users reported a positive attitude about e-cigarettes and negative attitude towards smoking, based on knowledge acquired beforehand. In general, e-cigarette users emphasized the positive aspects of e-cigarette devices and positive experiences, such as the variety of flavors and the adjustability of nicotine levels. 

### 3.3. Deliberation 

Many e-cigarette users consciously deliberated initiation of e-cigarette use versus continuation of cigarette smoking by using knowledge acquired and weighing up the benefits of e-cigarette use and disadvantages of cigarette smoking. For example, the personal health benefits of e-cigarette use, and the negative health issues for their social environment associated with secondhand smoking. They described this decision process of switching from cigarette smoking to e-cigarette use as a deliberation of benefits and disadvantages (quote 4):

*‘Switching from cigarettes to e-cigarettes […] it was a deliberate choice. I had an e-cigarette laying around and I tried it a few times. I liked the flavor and the satisfaction it gave me [throat hit from nicotine]. In the end I ordered a really nice one because I decided I was going to quit smoking. So yeah, it was a really deliberate choice…’*

*(#13_e-cigarette user)*

Many cigarette smokers and non-users reported that they had not contemplated using e-cigarettes. Deliberation was often limited to brief passive moments when they were confronted with an e-cigarette in their environment (quote 5).

*‘One of my friends had one [e-cigarette] and I thought: That might be a way to quit smoking.’*

*(#14_smoker)*

When asked to compare e-cigarette use initiation with cigarette smoking initiation, e-cigarette users and cigarette smokers agreed that cigarette smoking initiation was not a conscious or deliberate choice. Many cigarette smokers expressed they passively discovered themselves to be smokers months or years after they initiated smoking (quote 6).

*‘Everybody smoked, so I started too […] there wasn’t a point where I thought: OK, from now on I’m a smoker. All of a sudden, you just are one. Smoking is not a deliberate choice you make, something you really think about.’*

*(#1_smoker)*

In general, non-users did not show an interest in e-cigarettes or cigarettes. Pros and cons were not actively deliberated among non-users, but sometimes curiosity was reason enough to passively experiment with e-cigarettes or cigarettes. 

### 3.4. Information Need of E-Cigarettes 

When asked about their information need, all three groups stated they had several unanswered questions, such as, ‘Are e-cigarettes harmful?’, ‘What are the benefits of e-cigarette use compared to cigarette smoking?’, and ‘What are the negative long-term health effects of e-cigarette use?’. E-cigarette users, compared to smokers and non-users had more questions about product characteristics, such as ‘Are there results of quality tests of e-liquids available?’. Cigarette smokers and non-users wanted to know more about the successful quit attempts with e-cigarettes. They wanted to see a risk–benefit analysis of e-cigarette use versus smoking, and they wanted to know what the average duration of use was for e-cigarette use as a smoking cessation tool. All groups agreed that this information should be communicated to the public to facilitate smoking cessation for cigarette smokers. Many participants explained that there is a large body of information available, but that it is difficult to filter useful and correct information from the internet. 

### 3.5. Additional Aspects 

This section describes additional aspects participants mentioned that played a role in their decision about e-cigarette use.

#### 3.5.1. Risk Perception 

The risks of e-cigarettes versus cigarettes were discussed. E-cigarette users did not perceive any health risks of e-cigarette use (quote 7).

*‘So I thought: ‘it’s just vape. That can’t be really harmful for you or your lungs. So if I just vape, and I don’t do it every day […] that won’t be so wrong.’*

*(#4_e-cigarette user)*

Cigarette smokers stated that although they knew about the common risks of cigarettes, the specifics of how cigarettes causes smoking related diseases were not clear. They also perceived the health risks of cigarettes as a problem for the distant future, something to worry about later, when they were older. E-cigarette users tended to deliberate the risks and severity of cigarette smoking related diseases and acknowledged that the risks applied to them as former long-time cigarette smokers (quote 8):

*‘My grandfather smoked, like me, and you can see his health is getting worse and worse. He suffers from emphysema, I don’t want that. So I wanted to quit.’*

*(E-cigarette user_#26)*

While non-users were aware of the risks of cigarette smoking, the risks of e-cigarettes were unclear to them. Similar to e-cigarette users, they mentioned that if it is just vape, it did not seem harmful (quote 7). E-cigarette users perceived e-cigarette use compared to cigarette smoking not as a risk, but as a lifestyle (quote 9):

*‘I often say that besides a hobby it’s [e-cigarette use] also a lifestyle.’*

*(#21_e-cigarette user)*

Similar to e-cigarette users, cigarette smokers perceived cigarette smoking as an addiction, and they described triggers for the desire to smoke, such as alcohol consumption. Non-users perceived both e-cigarette use and cigarette smoking as addictive behaviors.

#### 3.5.2. Social Environment 

All groups described the importance of their social environment with regard to e-cigarette use and cigarette smoking. In the initiation of e-cigarette use, the social environment was an important factor for many e-cigarette users (quote 10).

*‘My mother had one [e-cigarette], so I tried it too.’*

*(#17_e-cigarette user)*

Several e-cigarette users further noted that social support for e-cigarette use from their social environment grew after initiation of use, which they described as a sense of belonging to an e-cigarette community (quote 11):

*‘It’s a community […] Very similar to bikers! You know how they all greet each other on the road? The vaping community is the same.’*

*(#21_e-cigarette user)*

In general, participants expressed it was more acceptable to smoke than to use e-cigarettes. While cigarette smokers and non-users categorized e-cigarette use as “weird”, cigarette smoking was still considered as acceptable by the several of participants and their social environment. When asked about the role of their social environment in their decision to initiate cigarette smoking compared to switching to e-cigarette use, cigarette smokers expressed that friends who smoked often influenced their decision (quote 12):

*‘One of my friends told me: ‘Try a cigarette!’ So I did, and I liked it and I continued smoking.’*

*(#15_smoker)*

Cigarette smokers did not describe the influence of friends as pressure, but they did describe the disappointment their parents expressed in response to finding out about their smoking behavior as embarrassing (quote 13):

*‘I was really afraid to tell my parents that I smoke […] they were so disappointed.’*

*(#11_smoker)*

Non-users viewed e-cigarette use and cigarette smoking not as a social activity, but as peer pressure and addiction, not a choice. Non-users felt strongly about parental disappointment related to cigarette smoking and noted this as a specific reason why they would not start smoking cigarettes or start using e-cigarettes.

#### 3.5.3. Trust in Information

Due to the large body of information available about e-cigarettes, it was difficult for participants to make sure the available information is correct. E-cigarette users and cigarette smokers mistrusted evidence-based information, and relied on anecdotal user experiences (quote 14):

*‘Researchers often don’t know what they are doing (#20_e-cigarette user) […] yes we need to know about the experiences of e-cigarette users.’*

*(#16_e-cigarette user)*

Unlike e-cigarette users and cigarette smokers, non-users explained they rather receive scientific information because they trust this information to be true.

## 4. Discussion

We explored the decision-making process of e-cigarette use initiation among adult e-cigarette users, adult cigarette smokers, and adolescent non-users. Compared to e-cigarette users, cigarette smokers and non-users did not make a conscious decision to refrain from e-cigarette use. They have acquired different information and have different attitudes. Several of e-cigarette users perceived the risks of cigarette smoking as personally relevant. They acquired information and formed an attitude about e-cigarettes. Finally, their knowledge, attitudes, pros, and cons were deliberated and a conscious decision was made to initiate e-cigarette use. Additionally, lack of information seeking, perception of risk of smoking related diseases, perception of risks and benefits of e-cigarette use compared to cigarette smoking, support to switch to e-cigarettes, a sense of belonging to e-cigarette users, and trust in information offered about e-cigarettes showed to be important factors for a participant in a decision-making process about e-cigarette use. 

A possible barrier in the decision-making process is the large body of contradicting information available and lack of trust in scientific evidence. Although e-cigarettes users appear knowledgeable, their reported knowledge did not match scientific consensus. We categorized this type of knowledge as beliefs, and these beliefs were primarily based on anecdotal user experiences found online. This finding matches research that shows that experienced e-cigarette users are eager to share their advice and experiences about switching with cigarette smokers [38]. Due to these beliefs, e-cigarette users perceived e-cigarettes as harmless. Research has showed that confirmation bias and online information may lead to misperceptions about the safety of e-cigarettes [39,40,41,42]. These beliefs and misperceptions about the harmfulness of e-cigarettes may hinder the process of making a conscious and informed decision about e-cigarette use initiation. Thus, there is a clear need for further research on targeted communication strategies to facilitate the conscious deliberation of relevant and evidence-based information about e-cigarette use. These strategies need to highlight advantages and disadvantages of switching to, or refraining from e-cigarette use, and correcting misperceptions about perceived risks and benefits of e-cigarettes [43]. This will emphasize the public and individual health benefits of e-cigarette use initiation for cigarette smokers and the disadvantages for non-users.

Cigarette smokers did not contemplate to switch to e-cigarettes, or consciously decide to continue smoking. Our results indicate that though cigarette smokers are aware of the health risks of cigarette smoking, they do not perceive the long-term health risks of smoking as personally relevant. Additionally, they perceive e-cigarettes as weird, smoking as a normal, social behavior [44], and, possibly due to their young age (19.8 (±3.2)), display an optimism bias about their ability to quit in time before serious health effects occur [30,45]. This optimism bias greatly diminishes cigarette smokers’ interest to acquire knowledge about the benefits and disadvantages of switching to exclusive e-cigarette use, and deliberate these pros and cons of e-cigarette use compared to smoking [30,45,46]. Low perceived susceptibility and severity of risks associated with cigarette smoking, a lack of knowledge, and a social environment in which e-cigarette use is considered weird and smoking is considered normal or encouraged, hinders smokers to deliberate the pros and cons of switching to e-cigarettes or smoking cessation. 

Similar to cigarette smokers, non-users did not actively deliberate e-cigarette use initiation. Adolescent non-users mentioned that if they were to start e-cigarette use, it would be out of curiosity. The risks of e-cigarette use were unclear to non-users, but because it was just vape, e-cigarette use was perceived as harmless. This lack of perceived personal risks of e-cigarette use may diminish the interest to acquire knowledge about the disadvantages of e-cigarette use. Without information seeking and information about advantages and disadvantages of e-cigarette use for non-users, no deliberation can take place [29]. A lack of deliberation of the risks associated with e-cigarette use, and curiosity about e-cigarette use may result in a tendency to initiate experimenting with e-cigarettes among some adolescents. Low perceived risk of e-cigarettes and a lack of knowledge hinder non-users to make a deliberate decision to refrain from e-cigarette use. 

This qualitative study shows that cigarette smokers and non-users did not consciously deliberate information about the risks and benefits of e-cigarettes use to make a decision to refrain from e-cigarette use initiation. The differences and similarities in the decision-making process of e-cigarette use among the different user groups raise a question. Namely, can insights into the conscious decision-making process of e-cigarettes users who switched from cigarette to e-cigarettes be used to stimulate cigarette smokers to consciously deliberate something they consider ‘weird’? Further research is necessary to investigate how cigarette smokers can be stimulated to consciously deliberate a switch to e-cigarettes. 

Because of the explorative nature of this study, the results are not meant to convey generalizability beyond the study population, and thus, these findings are limited to a specific geographic context and time. Respondents were asked to participate in a two-hour discussion, which may have attracted individuals more inclined to talk about cigarette smoking and e-cigarette use. Additionally, we asked e-cigarette users, cigarette smokers, and non-users to consider their decisions about initiation in retrospect, knowledge and attitudes may be different when first faced with the decision. Participants’ knowledge, attitudes, and deliberation process might be influenced by their level of education, and living in different areas of the Netherlands. Further research is necessary to explore the impact of education on a decision about e-cigarettes. 

## 5. Conclusions

To conclude, our exploration of the decision-making process of e-cigarette initiation identifies distinct differences between the decision-making process of current cigarette smokers, e-cigarette users, and non-users. Current e-cigarette users (former smokers) in this study made a conscious decision to switch from cigarettes to e-cigarettes, but current cigarette smokers and non-users did not contemplate or deliberate e-cigarette use initiation. E-cigarettes have a potential public and individual health benefit for cigarette smokers, as switching to exclusive e-cigarette use is less harmful than cigarette smoking. E-cigarette use is not harmless, initiation of e-cigarette use and cigarette smoking should thus be prevented among non-users. Several of the e-cigarette users deliberated personally relevant risks of cigarette smoking, which made them interested in switching to a less harmful alternative. These findings underline the importance to explore the possibility to learn from the decision-making process of e-cigarette users, in order to support cigarettes smokers with their decision about switching to e-cigarettes and non-users with their decision to refrain from e-cigarette use.

## Figures and Tables

**Table 1:** Demographic information of the study participants.

Demographics	E-Cigarette Users	Cigarette Smokers	Non-Users
**Age µ (±)**	31.8 (±12.2)	19.8 (±3.2)	15.2 (±1.1)
**Education n(%)**			
High	2 (8%)	7 (41%)	5 (22%)
Middle	15 (58%)	6 (35%)	8 (35%)
Low	9 (35%)	4 (24%)	10 (43%)
**Gender n(%)**			
Male	19 (73%	8 (47%)	8 (35%)
Female	7 (27%)	9 (53%)	15 (65%)

Note: Demographic information is displayed for each user group.

## Supplementary Materials

The following are available online at http://www.mdpi.com/1660-4601/16/4/624/s1, Table S1: Participant characteristics and alias, Table S2: Topic guide.

## References

[1] World Health Organization Tobacco Free Initiative (TFI). http://www.who.int/tobacco/communications/statements/eletronic_cigarettes/en/.

[2] Grana R., Benowitz N., Glantz S.A. (2014). E-cigarettes: A scientific review. Circulation.

[3] Glasser A.M., Collins L., Pearson J.L., Abudayyeh H., Niaura R.S., Abrams D.B., Villanti A.C. (2017). Overview of Electronic Nicotine Delivery Systems: A Systematic Review. Am. J. Prev. Med..

[4] Hajek P., Etter J.F., Benowitz N., Eissenberg T., McRobbie H. (2014). Electronic cigarettes: Review of use, content, safety, effects on smokers and potential for harm and benefit. Addiction.

[5] Pearson J.L., Amato M.S., Wang X., Zhao K., Cha S., Cohn A.M., Papandonatos G.D., Graham A.L. (2017). How US Smokers Refer to E-cigarettes: An Examination of User-Generated Posts from a Web-Based Smoking Cessation Intervention, 2008–2015. Nicotine Tob. Res..

[6] Visser W., Geraets L., Klerx W., Hernandez L., Croes E., Schwillens P., Cremers H., Bos P., Talhout R. (2015). The Health Risks of e-Cigarette Use.

[7] Xu Y., Guo Y., Liu K., Liu Z., Wang X. (2016). E-Cigarette Awareness, Use, and Harm Perception among Adults: A Meta-Analysis of Observational Studies. PLoS ONE.

[8] Feirman S.P., Lock D., Cohen J.E., Holtgrave D.R., Li T. (2016). Flavored Tobacco Products in the United States: A Systematic Review Assessing Use and Attitudes. Nicotine Tob. Res..

[9] Laverty A.A., Vardavas C.I., Filippidis F.T. (2016). Design and marketing features influencing choice of e-cigarettes and tobacco in the EU. Eur. J. Public Health.

[10] McRobbie H. (2017). Modelling the Population Health Effects of E-Cigarettes Use: Current Data Can Help Guide Future Policy Decisions. Nicotine Tob. Res..

[11] Stratton K., Kwan L.Y., Eaton D.L. (2018). Public Health Consequences of e-Cigarettes.

[12] Chen J., Bullen C., Dirks K. (2017). A Comparative Health Risk Assessment of Electronic Cigarettes and Conventional Cigarettes. Int. J. Envrion. Res. Public Health.

[13] Springvloet L., Bommele J., Willemsen M., van Laar M. (2018). Kerncijfers Roken 2017.

[14] World Health Organization Tobacco. http://www.who.int/mediacentre/factsheets/fs339/en/.

[15] Goniewicz M.L., Gawron M., Smith D.M., Peng M., Jacob P., Benowitz N.L. (2017). Exposure to Nicotine and Selected Toxicants in Cigarette Smokers Who Switched to Electronic Cigarettes: A Longitudinal Within-Subjects Observational Study. Nicotine Tob. Res..

[16] Ratajczak A., Feleszko W., Smith D.M., Goniewicz M. (2018). How close are we to definitively identifying the respiratory health effects of e-cigarettes?. Expert Rev. Respir. Med..

[17] Levy D.T., Borland R., Lindblom E.N., Goniewicz M.L., Meza R., Holford T.R., Yuan Z., Luo Y., O’Connor R.J., Niaura R. (2018). Potential deaths averted in USA by replacing cigarettes with e-cigarettes. Tob. Control.

[18] Burstyn I. (2014). Peering through the mist: Systematic review of what the chemistry of contaminants in electronic cigarettes tells us about health risks. BMC Public Health.

[19] Orr M.S. (2014). Electronic cigarettes in the USA: A summary of available toxicology data and suggestions for the future. Tob. Control.

[20] Soneji S., Barrington-Trimis J.L., Wills T.A., Leventhal A.M., Unger J.B., Gibson L.A., Yang J., Primack B.A., Andrews J.A., Miech R.A. (2017). Association Between Initial Use of e-Cigarettes and Subsequent Cigarette Smoking Among Adolescents and Young Adults: A Systematic Review and Meta-analysis. Jama Pediatr..

[21] Soneji S., Sung H.Y., Primack B., Pierce J.P., Sargent J. (2017). Problematic Assessment of the Impact of Vaporized Nicotine Product Initiation in the United States. Nicotine Tob. Res..

[22] Li Volti G., Polosa R., Caruso M. (2018). Assessment of E-cigarette impact on smokers: The importance of experimental conditions relevant to human consumption. Proc. Natl. Acad. Sci. USA.

[23] Soule E.K., Rosas S.R., Nasim A. (2016). Reasons for electronic cigarette use beyond cigarette smoking cessation: A concept mapping approach. Addict. Behav..

[24] Ayers J.W., Leas E.C., Allem J.P., Benton A., Dredze M., Althouse B.M., Cruz T.B., Unger J.B. (2017). Why do people use electronic nicotine delivery systems (electronic cigarettes)? A content analysis of Twitter, 2012–2015. PLoS ONE.

[25] Romijnders K., van Osch L., de Vries H., Talhout R. (2018). Perceptions and Reasons Regarding E-Cigarette Use among Users and Non-Users: A Narrative Literature Review. Int. J. Environ. Res. Public Health.

[26] Antognoli E., Koopman Gonzalez S., Trapl E., Cavallo D., Lavanty B., Lim R., Flocke S. (2018). Cigarettes, Little Cigars, and Cigarillos: Initiation, Motivation, and Decision-Making. Nicotine Tob. Res..

[27] Wadsworth E., Neale J., McNeill A., Hitchman S.C. (2016). How and Why Do Smokers Start Using E-Cigarettes? Qualitative Study of Vapers in London, UK. Int. J. Envrion. Res. Public Health.

[28] Marteau T.M., Dormandy E., Crockett R. (2005). Informed choice: Why measuring behaviour is important. Arch. Dis. Child.

[29] Van den Berg M., Timmermans D.R., ten Kate L.P., van Vugt J.M., van der Wal G. (2006). Informed decision making in the context of prenatal screening. Patient Educ. Couns..

[30] Baron J. (2007). Thinking and Deciding.

[31] Gray R.J., Hoek J., Edwards R. (2016). A qualitative analysis of ‘informed choice’ among young adult smokers. Tob. Control.

[32] Hoek J., Ball J., Gray R., Tautolo E.S. (2017). Smoking as an ‘informed choice’: Implications for endgame strategies. Tob. Control.

[33] Michie S., Dormandy E., Marteau T.M. (2003). Informed choice: Understanding knowledge in the context of screening uptake. Patient Educ. Couns..

[34] Chapman S., Liberman J. (2005). Ensuring smokers are adequately informed: Reflections on consumer rights, manufacturer responsibilities, and policy implications. Tob. Control.

[35] Dicicco-Bloom B., Crabtree B.F. (2006). The qualitative research interview. Med. Educ..

[36] Vaismoradi M., Turunen H., Bondas T. (2013). Content analysis and thematic analysis: Implications for conducting a qualitative descriptive study. Nurs. Health Sci..

[37] Neale J., Miller P., West R. (2014). Reporting quantitative information in qualitative research: Guidance for authors and reviewers. Addiction.

[38] Russell C., Dickson T., McKeganey N. (2018). Advice from Former-Smoking E-Cigarette Users to Current Smokers on How to Use E-Cigarettes as Part of an Attempt to Quit Smoking. Nicotine Tob. Res..

[39] Betsch C., Renkewitz F., Betsch T., Ulshofer C. (2010). The influence of vaccine-critical websites on perceiving vaccination risks. J. Health Psychol..

[40] Palminteri S., Lefebvre G., Kilford E.J., Blakemore S.J. (2017). Confirmation bias in human reinforcement learning: Evidence from counterfactual feedback processing. PLoS Comput. Biol..

[41] Glick M. (2017). Believing is seeing: Confirmation bias. J. Am. Dent. Assoc..

[42] Siegrist M., Stampfli N., Kastenholz H. (2008). Consumers’ willingness to buy functional foods. The influence of carrier, benefit and trust. Appetite.

[43] Compton J., Jackson B., Dimmock J.A. (2016). Persuading Others to Avoid Persuasion: Inoculation Theory and Resistant Health Attitudes. Frontiers in psychology.

[44] Lucherini M., Rooke C., Amos A. (2019). “They’re thinking, well it’s not as bad, I probably won’t get addicted to that. But it’s still got the nicotine in it, so...”: Maturity, Control, and Socializing: Negotiating Identities in Relation to Smoking and Vaping-A Qualitative Study of Young Adults in Scotland. Nicotine Tob. Res..

[45] Slovic P. (2000). What Does it Mean to Know a Cumulative Risk? Adolescents’ Perception of Short-term and Long-term Consequences of Smoking. J. Behav. Dec. Mak..

[46] Weinstein N.D., Marcus S.E., Moser R.P. (2005). Smokers’ unrealistic optimism about their risk. Tob. Control.

