# Supplementary material for: A Deliberate Choice? Exploring the Decision to Switch from Cigarettes to E-Cigarettes
Source: Int J Environ Res Public Health. 2019 Feb 20;16(4):624. doi: 10.3390/ijerph16040624 (PMC6406587; doi:10.3390/ijerph16040624)
Supplement: Supplementary file 1 [file ijerph-16-00624-s001.zip › 2019.02.19.Table S1_participant characteristics.doc]

Supplementary file A: Participant characteristics and alias

| Alias for participant | Age | Gender | Education level |
| --- | --- | --- | --- |
| 1_E-cigarette user | 46 | female | Low |
| 2_E-cigarette user | 37 | male | Low |
| 3_E-cigarette user | 22 | male | Low |
| 4_E-cigarette user | 18 | male | Middle |
| 5_E-cigarette user | 30 | male | Low |
| 6_E-cigarette user | 50 | male | Middle |
| 7_E-cigarette user | 42 | male | High |
| 8_E-cigarette user | 40 | male | Low |
| 9_E-cigarette user | 51 | male | Middle |
| 10_E-cigarette user | 43 | male | Middle |
| 11_E-cigarette user | 20 | female | Middle |
| 12_E-cigarette user | 24 | male | Middle |
| 13_E-cigarette user | 24 | male | Low |
| 14_E-cigarette user | 27 | male | Middle |
| 15_E-cigarette user | 48 | female | Middle |
| 16_E-cigarette user | 34 | male | Middle |
| 17_E-cigarette user | 31 | female | Low |
| 18_E-cigarette user | 31 | female | Middle |
| 19_E-cigarette user | 32 | male | High |
| 20_E-cigarette user | 48 | male | Middle |
| 21_E-cigarette user | 55 | male | Middle |
| 22_E-cigarette user | 58 | female | Middle |
| 23_E-cigarette user | 43 | female | Low |
| 24_E-cigarette user | 49 | male | Middle |
| 25_E-cigarette user | 55 | male | Middle |
| 26_E-cigarette user | 22 | male | Low |
| 1_Non-user | 13 | female | High |
| 2_Non-user | 15 | female | High |
| 3_Non-user | 17 | female | Middle |
| 4_Non-user | 14 | male | High |
| 5_Non-user | 16 | female | Low |
| 6_Non-user | 16 | male | Low |
| 7_Non-user | 15 | female | Low |
| 8_Non-user | 15 | female | Low |
| 9_Non-user | 16 | female | Low |
| 10_Non-user | 16 | female | Middle |
| 11_Non-user | 15 | female | Middle |
| 12_Non-user | 16 | male | Middle |
| 13_Non-user | 16 | male | Low |
| 14_Non-user | 16 | female | Low |
| 15_Non-user | 14 | female | Middle |
| 16_Non-user | 15 | female | Low |
| 17_Non-user | 16 | male | Low |
| 18_Non-user | 14 | male | High |
| 19_Non-user | 14 | female | Middle |
| 20_Non-user | 13 | female | middle |
| 21_Non-user | 16 | male | Middle |
| 22_Non-user | 17 | female | High |
| 23_Non-user | 16 | male | Low |
| 1_Smoker | 20 | male | High |
| 2_Smoker | 18 | female | Middle |
| 3_Smoker | 19 | female | Low |
| 4_Smoker | 29 | male | High |
| 5_Smoker | 20 | female | Low |
| 6_Smoker | 23 | female | Middle |
| 7_Smoker | 18 | male | Middle |
| 8_Smoker | 24 | female | Middle |
| 9_Smoker | 18 | male | High |
| 10_Smoker | 19 | male | High |
| 11_Smoker | 19 | female | High |
| 12_Smoker | 18 | female | High |
| 13_Smoker | 19 | male | Middle |
| 14_Smoker | 19 | male | Low |
| 15_Smoker | 18 | female | Middle |
| 16_Smoker | 18 | male | Low |
| 17_Smoker | 18 | female | High |
| **Note** the alias reflects the user group of the participant | | | |
