# Supplementary material for: A Deliberate Choice? Exploring the Decision to Switch from Cigarettes to E-Cigarettes
Source: Int J Environ Res Public Health. 2019 Feb 20;16(4):624. doi: 10.3390/ijerph16040624 (PMC6406587; doi:10.3390/ijerph16040624)
Supplement: Supplementary file 1 [file ijerph-16-00624-s001.zip › 2019.02.19.Table S2_Topic guide.doc]

Supplementary file B: Topic guide: exploring decision making process of e-cigarette use initiation

Note: All participants have to sign an informed consent before the start of the introduction.

Introduction by interviewer

Welcome! My name is … and this is …, who will be helping me with the discussion today. I work for … and I my research is about….

Today we are discussing e-cigarettes. Your opinion as user/smoker/non-user is very important. We would like to talk to you about e-cigarettes and this discussion will take approximately two hours, with a break. At the end of our discussion, we have time for general questions about e-cigarettes.

We would like to stress that there are no correct or wrong answers. Today is all about your experiences and your ideas. We will be hearing a lot of different ideas and experiences, so I would like to ask you to respect each other. Please listen carefully to each other and please let each other finish speaking.

This group discussion will be audio recorded. – *Turn on audio recording—*

All personal information will be made anonymous, and please remember that your participation is voluntary. This means that you can leave at all times, you do not have to explain yourself.

Are there any remaining questions before we start?

Introduction participants

Please introduce yourself shortly. Do you use e-cigarettes exclusively? Or do you also use cigarettes or something else? What do you enjoy doing?

- Make name tags
- General prompts
- What do you mean?
- Can you tell me more about that?
- What comes to mind?
- Can you explain that to me?
- Is there anything else that you can think of?

**Topic guide**: exploring decision making process of e-cigarette use initiation

| **Knowledge about e-cigarettes** | |
| --- | --- |
| What do you know about e-cigarettes? | |
| *Prompts* | Risks, batteries, taxes, different e-cigarettes, costs, health benefits |
| **Attitude** | |
| What did you think when you used it? | |
| What do you think of e-cigarettes? | |
| *Prompts* | How does it feel? Do you compare it to cigarette smoking? What do you think of the action of using e-cigarettes? |
| **Deliberation** | |
| Did you ever try an e-cigarette? | |
| When did you try an e-cigarette? | |
| How did you consider e-cigarette use? | |
| Who played a role in your decision? | |
| What played a role in your decision? | |
| How difficult was it to make a decision? | |
| *Prompts* | Did you consider using something else, like an NRT? Did you ask for advice? Did you go to a shop? Are you happy with your decision? Would you do it differently? What would have made it easier for you to make a decision about e-cigarette use?  *[use these prompts to link to next topic]* |
| **Information need** | |
| What information is important to make a decision about e-cigarette use? | |
| Where did you look for information about e-cigarettes? | |
| *Prompts* | Which information was important to you? Which source was important to you? How do you know how to trust information? Which information do you still miss? |
| **Closing** | |
| Is there anything we did not cover today that you find important related to e-cigarettes? | |
| Do you have any questions left? | |
| Note: due to the semi-structured protocol these questions are examples and may vary slightly in each focus group in both style and order. | |
